# Proteinase 3 (PR3)-Positive Perinuclear Anti-neutrophil Cytoplasmic Antibodies (pANCA) Vasculitis With Concurrent Invasive Aspergillosis Infection

**DOI:** 10.7759/cureus.78169

**Published:** 2025-01-29

**Authors:** David Smith, Venessa Herminie, Shiv Priya, Mario Zaghar A Shehata

**Affiliations:** 1 Adult Critical Care, St George's University Hospitals NHS Foundation Trust, London, GBR

**Keywords:** anca-associated vasculitis, eosinophilic granulomatosis with polyangiitis, granulomatosis with polyangiitis, invasive aspergillosis, proteinase 3 antibodies

## Abstract

Anti-neutrophil cytoplasmic antibodies (ANCA)-associated vasculitides are a group of rare diseases that cause inflammation of small to medium vessels. They comprise granulomatosis with polyangiitis (GPA), microscopic polyangiitis (MPA), and eosinophilic granulomatosis with polyangiitis (EGPA). As the signs and symptoms are varied, the diagnosis of vasculitis is complex and challenging; however, there are type-specific clinical presentations that can be factored into the existing classification criteria. The difficulty faced in diagnosis is compounded due to ANCA being present in other chronic illnesses. While ANCA vasculitis may be triggered by infection, it is rarely associated with invasive aspergillosis. We present a case of proteinase 3 (PR3)-positive vasculitis with concurrent invasive aspergillosis in a 68-year-old male in whom both the clinical presentation and immunochemical picture were atypical. However, he responded well to treatment with immunosuppression. This report highlights the complexities in the diagnosis of vasculitis and the limitations of existing diagnosis and inclusion criteria.

## Introduction

Anti-neutrophil cytoplasmic antibodies (ANCA)-associated vasculitides refer to a group of diseases causing inflammation and necrosis of small to medium-sized blood vessels as defined in Davidson's Principles and Practice of Medicine [1]. They consist of granulomatosis with polyangiitis (GPA), microscopic polyangiitis (MPA), and eosinophilic granulomatosis with polyangiitis (EGPA). Although they are diseases with distinct presentations (e.g., GPA typically affects the upper airways), there is considerable overlap between the three [2]. While ANCA-associated vasculitides are rare, however, global incidence rates have been on the rise over the past three decades. EGPA is the rarest form of ANCA-associated vasculitis, with a global incidence rate of 0.14-0.4 per million [3].

ANCAs are auto-antibodies directed against granules on the cytoplasm of neutrophils and lysosomes of monocytes. While the granules contain several proteins, the most clinically relevant antigens are myeloperoxidase (MPO) and proteinase 3 (PR3). A trigger such as infection leads to increased expression of MPO and PR3. The binding of ANCA to MPO and PR3 leads to the activation of neutrophils, their degranulation, and neutrophil cell death as well as the deployment of neutrophil extracellular traps (NETs). NETs lead to endothelial injury and thus extravascular inflammation. The increased exposure to NET components leads to further ANCA production and thus a vicious cycle ensues [4]. Typically, patients present with complaints of arthralgias, myalgias, or malaise. These symptoms may start several months before the onset of more specific ones, which may include haematuria, palpable purpura, diarrhoea, ear pain, or muffled sensation in the ears, nasal symptoms, sinus pain, wheezing, and rarely, haemoptysis [5]. Respiratory failure at presentation is atypical [6].

The diagnosis of vasculitis is complex, as diagnostic criteria are continuously evolving. The International Chapel Hill Consensus Conference 2012 offers a standard system for nomenclature; however, it is neither a classification system nor is it diagnostic [7]. Existing criteria such as the American College of Rheumatology/European Alliance of Associations of Rheumatology criteria for classification of ANCA-associated vasculitis, and the European Medicines Agency (EMA) algorithm use either a combination of clinical and laboratory parameters, or histological parameters only [8]. Blood samples can be sent, which may be tested using indirect Immunofluorescence (IIF) assay; many laboratories use this as a screening tool for ANCA-associated vasculitis. If the IIF assay is positive, an antigen-specific immunoassay such as ELISA then confirms the target antigen (MPO or PR3). IIF assay can differentiate between cytoplasmic ANCA (cANCA) or perinuclear-ANCA (pANCA) with pANCA being typically MPO-ANCA (as MPO is cationic and attaches to the negatively charged nuclear membrane) and cANCA typically PR3-ANCA as PR3 remains distributed in the cytoplasm. Imaging of the lungs and sinuses, as well as biopsy of lungs and kidneys can further aid in diagnosis and assist with differentiating the different types of ANCA vasculitis [9].

Once vasculitis has been confirmed, the first line treatment for life-threatening disease is cyclophosphamide with corticosteroids. Mycophenolate mofetil or methotrexate with corticosteroids are recommended in non-life-threatening cases. Once remission has been achieved, maintenance therapy with low-dose corticosteroids and methotrexate or mycophenolate mofetil or azathioprine or rituximab is recommended for at least two years [10]. ANCA can be positive in other conditions such as systemic lupus, cystic fibrosis, or chronic infections such as endocarditis [10]. Vasculitis may also be associated with hepatitis C, hepatitis B, and syphilis [10]. Therefore, these conditions must be ruled out before a definitive diagnosis of ANCA-associated vasculitis can be made. We present a case of PR3-positive vasculitis and concurrent invasive aspergillosis.

## Case presentation

A 68-year-old male patient presented to the emergency department in March 2024 with a six-week history of cough and new-onset shortness of breath that had initially begun during a trip to India. His past medical and surgical history included hypertension, gastroesophageal reflux disease, alcohol-related liver disease, latent tuberculosis diagnosed and treated with rifampicin, isoniazid, and pyridoxine six years previously, surgical resection of prostate cancer, and lumbar microdiscectomy. He was an ex-smoker, having stopped 15 years previously, and had a history of excessive alcohol consumption.

The patient had initially sought medical attention in February at a local hospital in India, where he had been treated with intravenous antibiotics (IV levofloxacin, IV piperacillin-tazobactam, and oral carbapenem). A chest CT scan at that time had revealed a right upper lobe nodule, right middle lobe ground-glass opacities, and left upper lobe bronchiectatic changes, without cavitation. The diagnosis at that time had been unclear; the patient reported being told the symptoms were due to pollution. On returning to the United Kingdom at the end of March, the patient had noticed a worsening of his cough, progressing from dry to producing greenish sputum, occasionally accompanied by haemoptysis. He also reported associated chest tightness and worsening shortness of breath. The patient denied any fever, weight loss, night sweats, and gastrointestinal or genitourinary symptoms.

**Figure 1:**
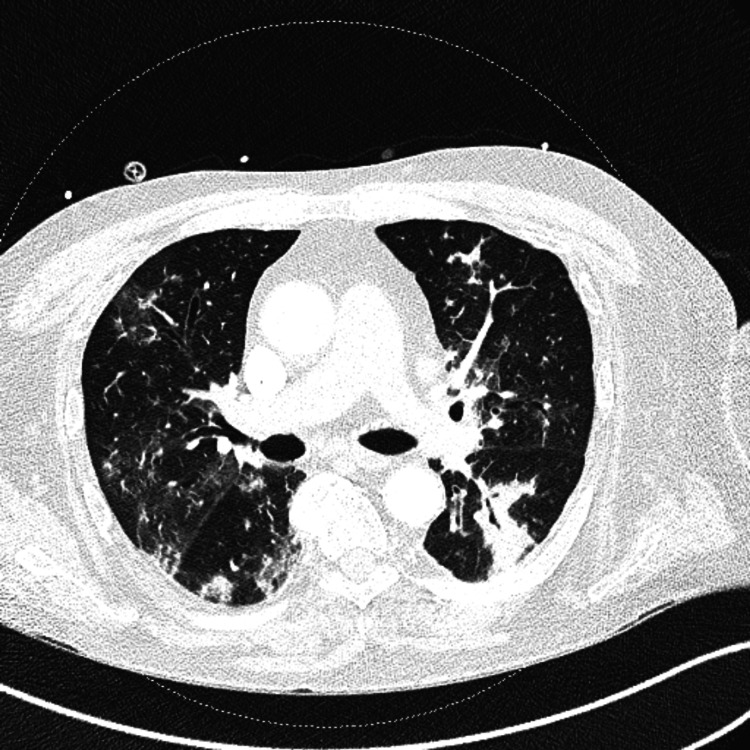
CT pulmonary angiogram showing florid patchy peri-bronchial opacification CT: computed tomography

On primary examination, the patient had a heart rate of 140 bpm in sinus rhythm, with an oxygen saturation of 95% on 3 L per minute of oxygen. Although oriented to time and place, he had mild confusion. His chest examination revealed scattered crepitations and wheeze, with normal heart sounds. He also had bilateral pedal eodema but showed no signs of deep vein thrombosis (DVT). A CT pulmonary angiography (CTPA) ruled out pulmonary embolism but demonstrated florid patchy peri-bronchial opacification, predominantly in the upper lobes, with cystic bronchiectasis and cavitation in the left upper lobe (Figure 1). There were also subtle calcified pleural plaques suggestive of prior asbestos exposure.

The initial impression was cavitating bacterial pneumonia with a differential diagnosis including tuberculosis. As the patient had a significant liver function derangement (Table 1) with mild coagulopathy (low prothrombin time and international normalised ratio; Table 1), there was also concern regarding possible decompensating cirrhosis.

**Table 1:** Blood results indicating worsening liver function and coagulopathy

Test	Results	Reference values
Alanine transaminase, U/L	55	0-52
Alkaline phosphatase, U/L	225	30-130
Gamma-glutamyl transferase, U/L	665	0-38
Prothrombin time, seconds	16.6	9-13
International normalised ratio	1.36	0.8-1.1

The patient was initially admitted to the acute medical ward and started on broad-spectrum antibiotics including meropenem, vancomycin, and amikacin. However, his condition did not improve, and his oxygen requirements increased. Therefore, due to a high suspicion of tuberculosis (despite negative TB investigations), he was started on a tailored anti-TB regimen given his cirrhosis. As his carbapenemase-producing Enterobacteriaceae (CPE) swab returned positive for New Delhi Metallo-beta-lactamase-producing Escherichia coli (NDM E. coli), he was commenced on ceftazidime/avibactam and aztreonam. He was also started on AmBisome (amphotericin B) for invasive aspergillosis (elevated initial beta-D-glucan levels; Table 2) and Candida albicans having been isolated in the sputum; however, this was stopped after three days.

**Table 2:** Initial beta-D-glucan level

Test	Results	Reference value
Initial beta-D-glucan, pg/mL	110	Less than 7

**Figure 2:**
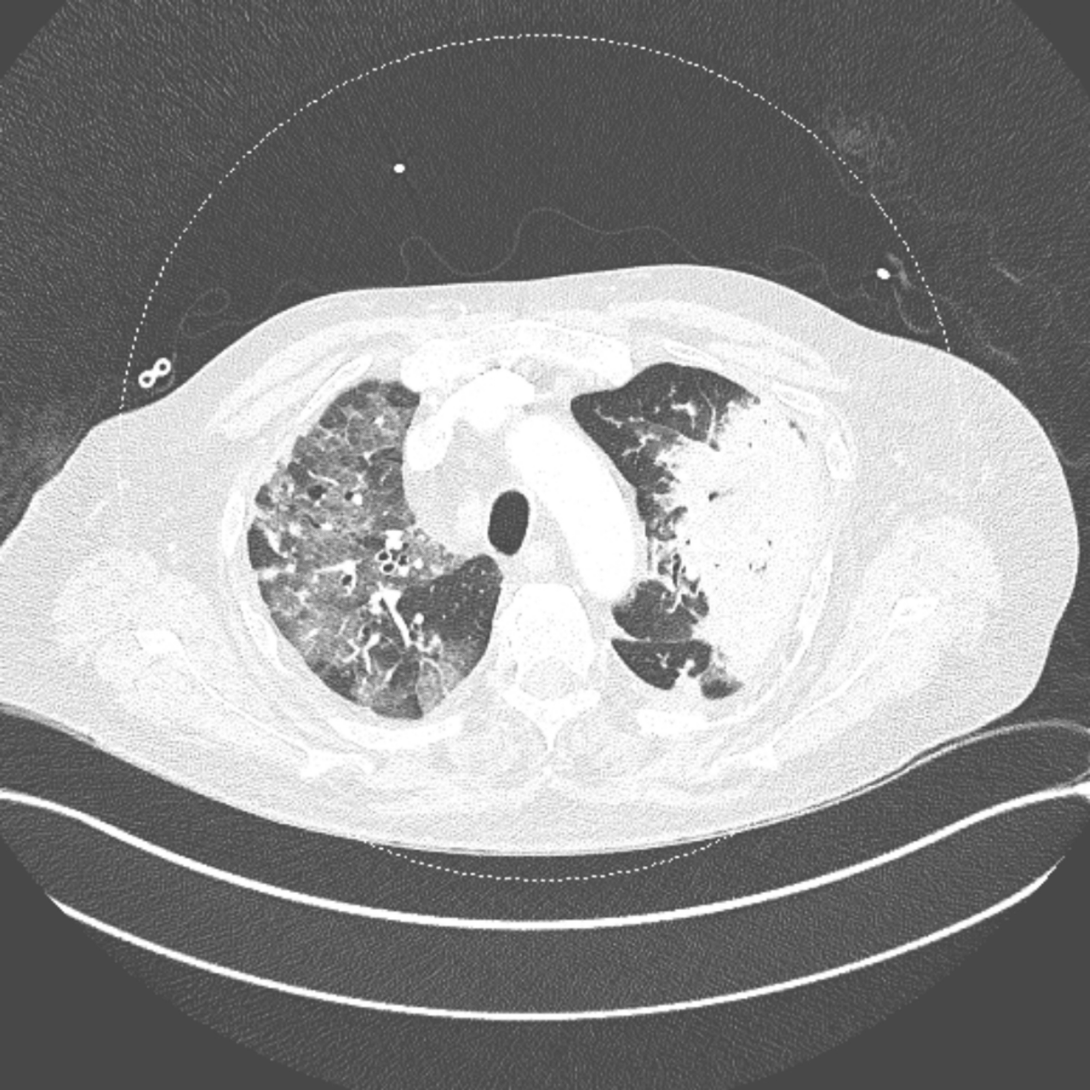
CT pulmonary angiogram two weeks after the admission showing worsening picture CT: computed tomography

A second CT scan, however, showed worsening cavitary parenchymal consolidation in the upper lobes and apical left lower lobe, and parenchymal ground glass opacity (Figure 2). The patient’s condition further deteriorated, and he was referred to the ICU for worsening type one respiratory failure. In the ICU, he was initially placed on high-flow nasal oxygen; however, a new hypercapnia and increased work of breathing warranted intubation and invasive ventilation.

As all cultures, GeneXpert test, and bronchoalveolar lavage (BAL) for tuberculosis returned negative, the TB treatment was discontinued. A low positive BAL galactomannan and a rising beta-D-glucan level (Table 3; repeat beta-D-glucan levels) led to the re-initiation of AmBisome (amphotericin B) and anidulafungin.

**Table 3:** Repeat beta-D-glucan level

Test	Results	Reference value
Repeat beta-D-glucan, pg/mL	323	Less than 7

A subsequent Aspergillus screen showed elevated Aspergillus precipitins IgG, with Aspergillus fumigatus identified in both BAL and sputum cultures. The patient was therefore initiated on posaconazole, and AmBisome was stopped. Oseltamivir was also started after influenza A was detected on a throat swab.

Despite these measures, the patient did not experience any clinical improvement. And, with the cessation of TB treatment, the cause for the cavitation seen on serial CT scans could not be explained; therefore an autoimmune screen was sent to rule out a possible vasculitis. The autoimmune panel revealed positive results for pANCA and PR3. Rheumatology evaluated the patient and expressed uncertainty regarding ANCA-associated vasculitis due to the atypical PR3/pANCA pattern, as it usually presents as a PR3/cANCA or MPO/pANCA pattern. However, the multidisciplinary team (MDT) agreed to trial pulsed methylprednisolone and cyclophosphamide, after which the patient's condition significantly ameliorated. Following a prolonged tracheostomy wean, he was discharged from the ICU after 34 days with cyclophosphamide ongoing as well as anidulafungin for aspergillosis.

**Figure 3:**
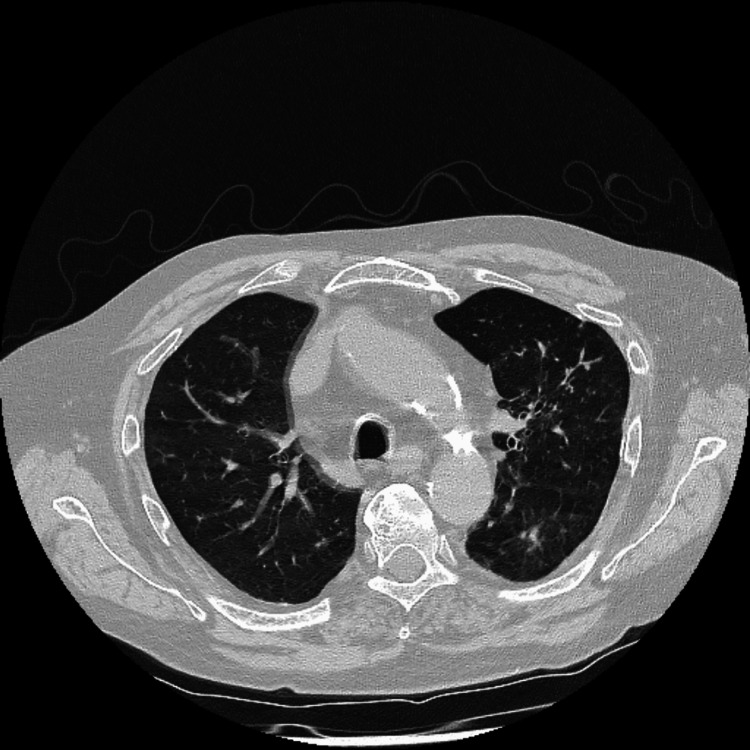
High-resolution CT scan of the lungs showing improving picture compared to previous scans CT: computed tomography

The patient was eventually discharged from the hospital after 44 days and continued to have cyclophosphamide as an outpatient. A repeat CT scan (Figure 3) in July demonstrated improvement, and serology showed a marked reduction in the ANCA titres as well as Aspergillus precipitins. At his follow-up review with his respiratory specialist in August, it was noted that the patient was continuing to drink 20 units of alcohol a day despite known liver cirrhosis. Although he had finished his cyclophosphamide regimen, he was not started on maintenance azathioprine and instead continued only on prednisolone.

## Discussion

There are three key learning points from this case report that warrant further discussion. Firstly, the uncertainty regarding the primary diagnosis before sending the initial vasculitic screen, especially the confounders of a history of previous TB and recent travel to India; secondly, the atypical presentation and immunochemical vasculitic picture and the wider implications this has on the existing criteria for vasculitis; and thirdly, the concurrent invasive aspergillosis in addition to the ANCA vasculitis.

At one point during his critical care admission, this patient was receiving concurrent treatment for TB, invasive aspergillosis, influenza, and bacterial pneumonia in the context of a confirmed CPE screen swab. However, these measures did not lead to any clinical improvement. In fact, with the cessation of TB treatment following multiple negative TB cultures and TB PCR, there was no obvious cause for the cavitation seen on serial CT scans. As the need to broaden the differential continued, a vasculitis screen was sent, appreciating that this picture of cavitating lung nodules can be seen in GPA (Table 4). The treatment strategies before the diagnosis of vasculitis were entirely reasonable given both the previous history of TB and recent travel to India. However, this highlights an unusual example of where the most likely diagnosis was not the actual diagnosis. Although beta-D glucan and galactomannan, in particular galactomannan in the BAL samples, indicated invasive aspergillosis, his condition did not improve on anidulafungin alone and he showed immediate improvement only after receiving treatment with cyclophosphamide and corticosteroids.

**Table 4:** GPA classification criteria according to ACR/EULAR 2022 guidelines A score of 5 or more is needed for the diagnosis of GPA ACR/EULAR: American College of Rheumatology/European Alliance of Associations for Rheumatology; GPA: granulomatosis with polyangiitis

ACR/EULAR 2022 GPA classification criteria	
Clinical criteria	Score
Nasal involvement: bloody discharge, ulcers, crusting, congestion, blockage, septal defect/perforation	+3
Cartilaginous involvement (inflammation of ear or nose cartilage, hoarse voice or stridor, endobronchial involvement, or saddle nose deformity)	+2
Conductive or sensorineural hearing loss	+1
-	-
Laboratory, imaging, and biopsy criteria	-
Positive test for cytoplasmic anti-neutrophil cytoplasmic antibodies (cANCA), or antiproteinase 3 (anti-PR3) antibodies	+5
Pulmonary nodules, mass, or cavitation on chest imaging	+2
Granuloma, extravascular granulomatous inflammation, or giant cells on biopsy	+2
Inflammation, consolidation, or effusion of the nasal/paranasal sinuses, or mastoiditis on imaging	+1
Pauci-immune glomerulonephritis on biopsy	+1
Positive test for perinuclear anti-neutrophil cytoplasmic antibodies (pANCA) or myeloperoxidase (anti-MPO) antibodies	-1
Blood eosinophil count >1 x 10^9^/litre	-4

Secondly, the patient had an atypical immunochemical picture and atypical clinical presentation that did not satisfy the current classification criteria. Traditionally, vasculitis was classified according to vessel size. With the advent of ANCA testing and modern imaging techniques, vasculitis could further be classified according to antibodies involved in the pathogenesis of the disease. The International Chapel Hill Consensus Conference on the Nomenclature of Systemic Vasculitides was convened to refine the nomenclature and standardise names and definitions as appropriate. The three main categories of dominant vessel pattern involvement are large vessels, medium vessels, and small vessels [7]. There is considerable overlap, however, and any vessel can be involved in any of the three categories. Small vessel vasculitis may be further divided into immune complex small vessel vasculitis, anti-GBM (glomerular basement membrane) disease, and ANCA-associated small vessel vasculitis.

The ANCA-associated small vessel vasculitis consists of three distinct phenotypes: GPA, MPA, and EGPA [7]. In 2002, the American College of Rheumatology and the European Alliance of Associations of Rheumatology (ACR/EULAR 2022) created classification criteria for the different types of ANCA-associated vasculitis to further aid in the diagnosis of these illnesses. These criteria are point-based and include both clinical and laboratory parameters that are distinct for GPA (Table 4), MPA, and EGPA (Table 5). Based on ACR/EULAR 2022, the classic autoimmune panel findings involve a cANCA/PR3 pairing or a pANCA/MPO. Whereas, in this case, the patient had a pANCA/PR3 pattern. This explains the potential for uncertainty regarding the diagnosis from Rheumatology described above and also highlights not only the atypical clinical presentation (in so far as no clinical criteria were present) but also the unusual immunological pattern. The instance of non-classifiable vasculitides has been discussed in the literature previously, with Rathmann et al. reporting that approximately 4% of patients were unclassifiable [8].

**Table 5:** EGPA classification criteria as per ACR/EULAR 2022 guidelines A score of 6 or more is needed for the diagnosis of EGPA ACR/EULAR: American College of Rheumatology/European Alliance of Associations for Rheumatology; EGPA: eosinophilic granulomatosis with polyangiitis

ACR/EULAR 2022 EGPA classification criteria	
Clinical criteria	Score
Obstructive airway disease	+3
Nasal polyps	+3
Mononeuritis multiplex	+1
	-
Laboratory and biopsy criteria	-
Blood eosinophil count ≥1x10^9^/litre	+5
Extravascular eosinophilic-predominant inflammation on biopsy	+2
Positive test for cytoplasmic anti-neutrophil cytoplasmic antibodies (cANCA) or antiproteinase 3 (anti-PR3) antibodies	-3
Haematuria	-1

Finally, ANCA-associated vasculitis is rarely associated with systemic aspergillosis; a Medline search revealed only a handful of case studies describing this association [11,12,13]. Infections are known to be triggers; 63% of patients with GPA chronically carry the common bacteria Staphylococcus aureus in their noses, and nasal inflammation is common in GPA [14]. The papers that describe an association between aspergillosis and vasculitis only speak of chronic allergic bronchopulmonary aspergillosis (ABPA) and do not describe an association with acute invasive aspergillosis [11,12,13]. Aspergillosis is generally associated with immunocompromised individuals, patients who have had TB in the past, asthmatics, and cystic fibrosis [15]. Acute invasive aspergillosis presents with cough, dyspnoea, and fever unresponsive to antibiotics [15]. Symptoms that are not unlike those described for vasculitis. Furthermore, the patient's eosinophilic count was raised, which could be due to aspergillosis or EGPA [16]. The patient's symptoms fit some of the criteria for the diagnosis of ANCA-associated vasculitis (PR3 positivity with ANCA antibodies, lung cavitations, and obstructive air disease on presentation along with eosinophilia - 2022 ACR/EULAR); however, those were not sufficient for a definitive diagnosis based on current definitions [17].

## Conclusions

We discussed the case of a 68-year-old male with an atypical immunological picture of pANCA and PR3 vasculitis and concurrent invasive aspergillosis who responded to immunotherapy despite fulfilling none of the accepted diagnostic clinical criteria for systemic vasculitis. The diagnosis of the vasculitis was further complicated by a strong clinical suspicion for an alternative diagnosis given his previous TB infection, recent travel to India, and a CPE-positive swab. This report highlights the difficulty encountered in triaging a potential vasculitis diagnosis and the limitations of the current diagnosis and inclusion criteria.
